# Direct Dried Stool Sampling on Filter Paper for Molecular Analyses of Cholera

**DOI:** 10.4269/ajtmh.16-0246a

**Published:** 2016-07-06

**Authors:** Stanislas Rebaudet, Sandra Moore, Anne-Cécile Normand, Lamine Koivogui, Eric Garnotel, Amara Jambai, Renaud Piarroux

**Affiliations:** ^1^ Parasitology Laboratory, Assistance Publique—Hôpitaux de Marseille, Marseille, France and UMR MD3, Aix-Marseille University, Marseille, France. E-mail: stanreb@gmail.com.; ^2^ UMR MD3, Aix-Marseille University, Marseille, France. E-mail: sandy.moore17@gmail.com.; ^3^ Parasitology Laboratory, Assistance Publique—Hôpitaux de Marseille, Marseille, France. E-mail: Anne-Cecile.Normand@ap-hm.fr.; ^4^ Institut National de Santé Publique, Ministère de la Santé Publique et de l'Hygiène Publique, Conakry, Republic of Guinea. E-mail: koivoguil@gmail.com.; ^5^ Laboratoire de Biologie, Hôpital d'instruction des armées (HIA) Alphonse Laveran, Marseille, France. E-mail: egarnotel@gmail.com.; ^6^ Disease Prevention and Control, Ministry of Health and Sanitation, Freetown, Sierra Leone. E-mail: amarajambai@yahoo.com.; ^7^ Parasitology Laboratory, Assistance Publique—Hôpitaux de Marseille, Marseille, France and UMR MD3, Aix-Marseille University, Marseille, France. E-mail: Renaud.PIARROUX@ap-hm.fr.

Dear Sir:

We read with great interest the recent article by Debes and others^1^ concerning simplified cholera surveillance methods performed in the Far North Region of Cameroon in 2013–2014. The authors described the novel use of filter paper to preserve DNA specimens for polymerase chain reaction (PCR) confirmation of cholera. In another recent article,^2^ they used the same sampling method to genotype *Vibrio cholerae* using multiple loci variable number tandem repeat (VNTR) analysis (MLVA), in the same resource-constrained area. In both studies, stool specimens were initially enriched for 6–8 hours in alkaline peptone water (APW). One to two drops of the enrichment were subsequently aliquoted onto Whatman 903 Protein Saver Cards (GE Healthcare Limited, Chalfont St.Giles, UK) and allowed to air-dry.

While investigating the origin of a cholera epidemic in the Republic of Guinea^3^ in September 2012, we experimented direct dried stool sampling on filter paper in the neighboring Sierra Leone, without prior enrichment in APW. Indeed, analysis of surveillance data and field investigations suggested a recent importation of cholera from Sierra Leone. However, lacking laboratory capacities in this country impaired the inclusion of samples for genetic comparison with Guinean culture isolates.^4^ Dried blood or saliva specimens on filter paper are relatively easy to collect, store, and transport, as they are not subject to United Nations biosafety regulations for transport of category B infectious substances.^5^ Moreover, direct PCR detection of *V. cholerae* on stool samples has been shown to represent a successful diagnosis method for cholera.^6^

With official authorization of Sierra Leonean health authorities, 17 suspected cholera patients were thus anonymously sampled in cholera treatment units in several coastal districts (Supplemental Figure 1). One or two drops of their watery diarrhea were directly collected from nonchlorinated buckets below cholera cots and then deposited onto each sample area of Whatman FTA Elute Micro Cards (GE Healthcare Limited, Chalfont St. Giles, UK). Each sample was air-dried for approximately 5 minutes in a shaded area, sealed with a Desiccant Packet (GE Healthcare Limited, Chalfont St. Giles, UK) into individual Multi-Barrier Pouches (GE Healthcare Limited, Chalfont St. Giles, UK), transported to Marseille, France, and stored at ambient temperature. For technical reasons, DNA extraction was performed only in June 2015. Using a scalpel, a 1-cm^2^ area of the filter paper was removed and suspended in 500 μL NucliSENS^®^ easyMAG^®^ lysis buffer (bioMérieux, Marcy l'Etoile, France) overnight at 4°C. The DNA was then extracted using a NucliSENS^®^ easyMAG^®^ platform (bioMérieux, Marcy l'Etoile, France) as previously described,^7^ and the supernatants were stored at −20°C for downstream PCR assays. To check for conserved DNA, a 16S ribosomal DNA PCR was performed as previously described.^8^ Six previously described VNTRs^7^ were independently genotyped for each sample. All primer pairs were verified to be specific for *V. cholerae* using National Center for Biotechnology Information (NCBI) Basic Local Alignment Search Tool (BLAST), and one primer set was located within the cholera toxin A subunit promoter region. MLVA results of the Sierra Leonean samples were included within the genotype panel of 35 Guinean isolates isolated via conventional culture in February–September 2012 (Supplemental Figure 1) and genotyped using the same method.^7^

The presence of amplifiable bacterial DNA was confirmed by 16S PCR in all 17 filter paper samples (Supplemental Table 1). Toxigenic *V. cholerae* was confirmed and MLVA genotyping was completed for nine of them. All VNTR-specific PCRs were negative for the eight remaining samples, which suggest that the corresponding patients may have been affected by noncholera acute watery diarrhea. The panel of nine genotyped samples from Sierra Leone displayed four distinct MLVA types (1, 5, 6, and 7). Together with the samples from Guinea, these MLVA types formed a single clonal complex of 13 closely related MLVA types (Supplemental Figure 1). Two MLVA types (5 and 6) were common to both Sierra Leonean and Guinean samples (Supplemental Figure 1).

We thus confirm that filter paper is a very convenient, inexpensive, and efficient tool to sample suspected cholera cases for delayed molecular studies, including *V. cholerae* MLVA genotyping, even after several years of storage at room temperature. Moreover, we demonstrate that prior stool enrichment in APW is not necessary to perform specific *V. cholerae* PCRs, which renders sampling even easier. Further experiments are required to determine whether this method of DNA collection and conservation is sufficient for *V. cholerae* whole-genome single nucleotide polymorphism–based phylogenetics.

In addition, MLVA genotyping of these cholera filter paper samples further bolsters the initial conclusion that Sierra Leone and the Republic of Guinea were affected in 2012 by the same transborder cholera epidemic,^3^ which was genetically related to outbreaks in Togo between 2010 and 2012.^7^

Such genotyping results can provide valuable insight to optimize control and prevention strategies. Direct stool sampling on filter papers should therefore be included in the rapid response package to investigate cholera epidemics, especially when *V. cholerae* culture and strain storage facilities are not available or biosafety shipping to specialized genotyping laboratories is too complicated.

## Supplementary Material

Supplemental Figures.

## References

[1] Debes AK, Ateudjieu J, Guenou E, Ebile W, Sonkoua IT, Njimbia AC, Steinwald P, Ram M, Sack DA (2016). Clinical and environmental surveillance for *Vibrio cholerae* in resource constrained areas: application during a 1-year surveillance in the far north region of Cameroon. Am J Trop Med Hyg.

[2] Debes AK, Ateudjieu J, Guenou E, Lopez AL, Bugayong MP, Retiban PJ, Garrine M, Mandomando I, Li S, Stine OC, Sack DA (2016). Evaluation in Cameroon of a novel, simplified methodology to assist molecular microbiological analysis of *V. cholerae* in resource-limited settings. PLoS Negl Trop Dis.

[3] Rebaudet S, Mengel MA, Koivogui L, Moore S, Mutreja A, Kande Y, Yattara O, Sarr Keita V, Njanpop-Lafourcade B-M, Fournier P-E, Garnotel E, Keita S, Piarroux R (2014). Deciphering the origin of the 2012 cholera epidemic in Guinea by integrating epidemiological and molecular analyses. PLoS Negl Trop Dis.

[4] Chattaway MA, Kamara A, Rhodes F, Kaffeta K, Jambai A, Alemu W, Islam MS, Freeman MM, Welfare W, Harding D, Samba AF, Abu M, Kamanda S, Grant K, Jenkins C, Nair S, Connell S, Siorvanes L, Desai S, Allen C, Frost M, Hughes D, Jeffrey Z, Gill N, Salter M (2014). Establishing an enteric bacteria reference laboratory in Sierra Leone. J Infect Dev Ctries.

[5] UNECE (2013). UN Recommendations on the Transport of Dangerous Goods: Model Regulations.

[6] Varela P, Pollevick GD, Rivas M, Chinen I, Binsztein N, Frasch AC, Ugalde RA (1994). Direct detection of *Vibrio cholerae* in stool samples. J Clin Microbiol.

[7] Moore S, Miwanda B, Sadji AY, Thefenne H, Jeddi F, Rebaudet S, de Boeck H, Bidjada B, Depina J-J, Bompangue D, Abedi AA, Koivogui L, Keita S, Garnotel E, Plisnier P-D, Ruimy R, Thomson N, Muyembe J-J, Piarroux R (2015). Relationship between distinct African cholera epidemics revealed via MLVA haplotyping of 337 *Vibrio cholerae* isolates. PLoS Negl Trop Dis.

[8] Paster BJ, Boches SK, Galvin JL, Ericson RE, Lau CN, Levanos VA, Sahasrabudhe A, Dewhirst FE (2001). Bacterial diversity in human subgingival plaque. J Bacteriol.

