# Supplementary material for: Direct Dried Stool Sampling on Filter Paper for Molecular Analyses of Cholera
Source: Am J Trop Med Hyg. 2016 Jul 6;95(1):251–2. doi: 10.4269/ajtmh.16-0246a (PMC4944700; doi:10.4269/ajtmh.16-0246a)
Supplement: Supplementary file 1 [file SD6.pdf]

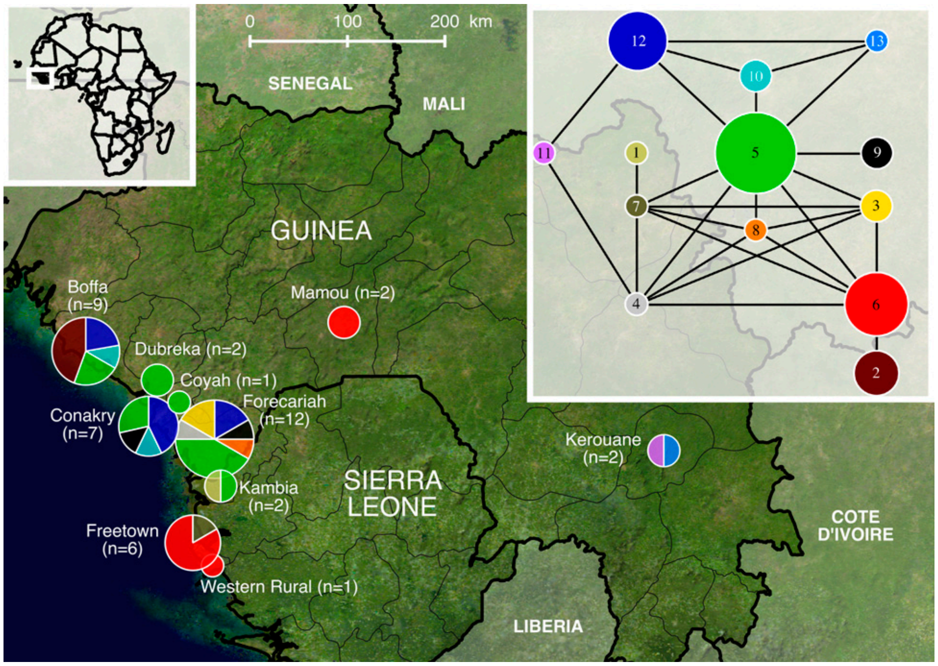


Supplemental Figure 1. Multiple loci variable number tandem repeat (VNTR) analysis (MLVA) types, relatedness, and spatial distribution of 44 *Vibrio cholerae* clinical isolates from the 2012 cholera epidemic in Sierra Leone and Guinea. Nine samples were collected in Sierra Leone in September 2012 using dried spots of stool on filter papers, and 35 isolates were sampled via conventional stool culture in Guinea between February and September 2012. Six VNTRs (VC1, VC4, VC5, VC9, LAV6, and VCMS12) were genotyped as previously described.[7] Thirteen MLVA types were identified. On the MLVA network, each MLVA type is represented by a node and is identified by a number. The size of the nodes reflects the number of isolates with each MLVA type. The solid lines indicate the single locus variants. On the map, pie charts indicate the spatial distribution of MLVA types in both countries. MLVA network was performed using RStudio version 0.98.994 for Mac (http://www.rstudio.com/, accessed March 7, 2016) with R version 3.1.1 (http://www.r-project.org/, accessed March 7, 2016) and igraph package (https://cran.r-project.org/web/packages/igraph/index.html, accessed March 7, 2016). The map was drawn using QGIS v2.12.1-Lyon (http://www.qgis.org/en/site/, accessed March 7, 2016).

Supplemental Table 1

List of clinical cholera isolates included in this study

| Sampling | | | | | MLVA | | | | | | |
|---|---|---|---|---|---|---|---|---|---|---|---|
| Date | Country | Province/Prefecture | Sampling method | 16S rDNA PCR | VC1 | VC4 | VC5 | VC9 | LAV6 | VCMS12 | MLVA genotype |
| September 2012 | Sierra Leone | Kambia | Filter paper | Positive | 185 | 238 | 188 | 188 | 299 | 272 | 1 |
| September 2012 | Sierra Leone | Kambia | Filter paper | Positive | 185 | 243 | 188 | 188 | 281 | 272 | 5 |
| September 2012 | Sierra Leone | Kambia | Filter paper | Positive | ND | ND | ND | ND | ND | ND | |
| September 2012 | Sierra Leone | Kambia | Filter paper | Positive | ND | ND | ND | ND | ND | ND | |
| September 2012 | Sierra Leone | Kambia | Filter paper | Positive | ND | ND | ND | ND | ND | ND | |
| September 2012 | Sierra Leone | Freetown | Filter paper | Positive | 185 | 243 | 188 | 188 | 287 | 272 | 6 |
| September 2012 | Sierra Leone | Port Loko | Filter paper | Positive | ND | ND | ND | ND | ND | ND | |
| September 2012 | Sierra Leone | Freetown | Filter paper | Positive | 185 | 243 | 188 | 188 | 287 | 272 | 6 |
| September 2012 | Sierra Leone | Freetown | Filter paper | Positive | ND | ND | ND | ND | ND | ND | |
| September 2012 | Sierra Leone | Freetown | Filter paper | Positive | 185 | 243 | 188 | 188 | 287 | 272 | 6 |
| September 2012 | Sierra Leone | Freetown | Filter paper | Positive | ND | ND | ND | ND | ND | ND | |
| September 2012 | Sierra Leone | Freetown | Filter paper | Positive | 185 | 243 | 188 | 188 | 287 | 272 | 6 |
| September 2012 | Sierra Leone | Western Rural | Filter paper | Positive | 185 | 243 | 188 | 188 | 287 | 272 | 6 |
| September 2012 | Sierra Leone | Freetown | Filter paper | Positive | 185 | 243 | 188 | 188 | 287 | 272 | 6 |
| September 2012 | Sierra Leone | Freetown | Filter paper | Positive | ND | ND | ND | ND | ND | ND | |
| September 2012 | Sierra Leone | Freetown | Filter paper | Positive | ND | ND | ND | ND | ND | ND | |
| September 2012 | Sierra Leone | Freetown | Filter paper | Positive | 185 | 243 | 188 | 188 | 299 | 272 | 7 |
| February 2012 | Guinea | Forecariah | culture | NA | 185 | 243 | 188 | 188 | 281 | 272 | 5 |
| February 2012 | Guinea | Forecariah | culture | NA | 185 | 243 | 188 | 188 | 281 | 272 | 5 |
| March 2012 | Guinea | Forecariah | culture | NA | 185 | 243 | 188 | 188 | 311 | 272 | 8 |
| March 2012 | Guinea | Forecariah | culture | NA | 185 | 243 | 188 | 188 | 281 | 272 | 5 |
| April 2012 | Guinea | Conakry | culture | NA | 185 | 243 | 188 | 188 | 281 | 272 | 5 |
| March 2012 | Guinea | Boffa | culture | NA | 185 | 249 | 188 | 188 | 281 | 272 | 10 |
| March 2012 | Guinea | Boffa | culture | NA | 185 | 243 | 188 | 188 | 281 | 272 | 5 |
| March 2012 | Guinea | Boffa | culture | NA | 185 | 243 | 188 | 188 | 281 | 272 | 5 |
| August 2012 | Guinea | Dubreka | culture | NA | 185 | 243 | 188 | 188 | 281 | 272 | 5 |
| August 2012 | Guinea | Dubreka | culture | NA | 185 | 243 | 188 | 188 | 281 | 272 | 5 |
| September 2012 | Guinea | Boffa | culture | NA | 185 | 243 | 188 | 181 | 287 | 272 | 2 |
| August 2012 | Guinea | Boffa | culture | NA | 185 | 255 | 188 | 188 | 281 | 272 | 12 |
| August 2012 | Guinea | Boffa | culture | NA | 185 | 243 | 188 | 181 | 287 | 272 | 2 |
| September 2012 | Guinea | Kerouane | culture | NA | 185 | 255 | 188 | 188 | 275 | 272 | 11 |
| September 2012 | Guinea | Kerouane | culture | NA | 185 | 272 | 188 | 188 | 281 | 272 | 13 |
| March 2012 | Guinea | Forecariah | culture | NA | 185 | 243 | 188 | 195 | 281 | 272 | 9 |
| March 2012 | Guinea | Conakry | culture | NA | 185 | 243 | 188 | 195 | 281 | 272 | 9 |
| September 2012 | Guinea | Boffa | culture | NA | 185 | 243 | 188 | 181 | 287 | 272 | 2 |
| July 2012 | Guinea | Conakry | culture | NA | 185 | 255 | 188 | 188 | 281 | 272 | 12 |
| July 2012 | Guinea | Conakry | culture | NA | 185 | 255 | 188 | 188 | 281 | 272 | 12 |
| May 2012 | Guinea | Mamou | culture | NA | 185 | 243 | 188 | 188 | 287 | 272 | 6 |
| May 2012 | Guinea | Mamou | culture | NA | 185 | 243 | 188 | 188 | 287 | 272 | 6 |
| July 2012 | Guinea | Conakry | culture | NA | 185 | 255 | 188 | 188 | 281 | 272 | 12 |
| April 2012 | Guinea | Forecariah | culture | NA | 185 | 243 | 188 | 188 | 275 | 272 | 4 |
| July 2012 | Guinea | Forecariah | culture | NA | 185 | 243 | 188 | 188 | 269 | 272 | 3 |
| August 2012 | Guinea | Coyah | culture | NA | 185 | 243 | 188 | 188 | 281 | 272 | 5 |
| July 2012 | Guinea | Forecariah | culture | NA | 185 | 243 | 188 | 188 | 269 | 272 | 3 |
| August 2012 | Guinea | Boffa | culture | NA | 185 | 255 | 188 | 188 | 281 | 272 | 12 |
| August 2012 | Guinea | Forecariah | culture | NA | 185 | 243 | 188 | 188 | 281 | 272 | 5 |
| September 2012 | Guinea | Forecariah | culture | NA | 185 | 255 | 188 | 188 | 281 | 272 | 12 |
| August 2012 | Guinea | Boffa | culture | NA | 185 | 243 | 188 | 181 | 287 | 272 | 2 |
| September 2012 | Guinea | Forecariah | culture | NA | 185 | 243 | 188 | 188 | 281 | 272 | 5 |
| August 2012 | Guinea | Forecariah | culture | NA | 185 | 255 | 188 | 188 | 281 | 272 | 12 |
| June 2012 | Guinea | Conakry | culture | NA | 185 | 249 | 188 | 188 | 281 | 272 | 10 |
| June 2012 | Guinea | Conakry | culture | NA | 185 | 243 | 188 | 188 | 281 | 272 | 5 |

MLVA = multiple loci variable number tandem repeat analysis; PCR = polymerase chain reaction; rDNA = ribosomal DNA; ND = no data; NA = not applicable.
